# Comparative Evaluation of Microscopy, Rapid Diagnostic Tests, and Polymerase Chain Reaction (PCR) for Malaria Diagnosis in Nigerian Children

**DOI:** 10.7759/cureus.73739

**Published:** 2024-11-15

**Authors:** Oyindamola G Osun, Abdulmalik S Ahmed, Salma A Suliman, Adedolapo B Olorunfemi, Bolaji N Thomas, Olusola Ojurongbe

**Affiliations:** 1 Department of Medical Microbiology and Parasitology, Ladoke Akintola University of Technology (LAUTECH), Ogbomoso, NGA; 2 Department of Life Sciences, Kano State Polytechnic, Kano, NGA; 3 Institute of Endemic Diseases, University of Khartoum, Khartoum, SDN; 4 Humboldt Research Hub-Center for Emerging and Reemerging Infectious Diseases, Ladoke Akintola University of Technology (LAUTECH), Ogbomoso, NGA; 5 Department of Biomedical Sciences, Rochester Institute of Technology, Rochester, USA

**Keywords:** malaria diagnosis, malaria in children, microscopy, plasmodium falciparum, polymerase chain reaction, rapid diagnostic test

## Abstract

Background

Malaria, a persistent public health issue in Nigeria, particularly among children, is often complicated by misdiagnosis, hindering effective treatment and control. The global adoption of rapid diagnostic tests (RDTs) for malaria has significantly improved management. This study, therefore, compares the diagnostic performance of microscopy, RDT, and polymerase chain reaction (PCR) for Plasmodium falciparum detection in children in Kano state, Nigeria, providing crucial insights for effective control and elimination.

Methods

Capillary blood samples and dried blood spots (DBS) were collected from 200 febrile children in a selected health center in Kano and systematically tested using RDT, microscopy, and nested polymerase PCR. The sensitivity and specificity for each diagnostic method were calculated, with nested PCR as the reference to assess diagnostic accuracy. Structured questionnaires were used to obtain information on possible risk factors. Data were analyzed using univariate, bivariate, and multivariate analysis, with p<0.05 considered significant.

Results

Out of the 200 children enrolled in the study, 112 (56%) tested positive by microscopy, 60 (30%) by RDT, and 70 (35%) by PCR. Using PCR (n=70) as the reference test, microscopy demonstrated a sensitivity of 73% and a specificity of 53%, while the RDT exhibited a lower sensitivity of 27% but a higher specificity of 68%. False positivity rates of 22 (31.5%) for RDT and 33 (46.9%) for microscopy were observed. Regarding false negatives, RDT had a much higher rate of 51 (72.9%) than microscopy of 19 (27.1%).

Conclusion

The study divulges the limitations of RDT in malaria detection, particularly its low sensitivity. With its higher sensitivity, microscopy also showed a significant false-positive rate. The study suggests a practical solution combining RDT and microscopy for routine malaria diagnosis, which could significantly improve diagnostic accuracy. More ongoing research is needed to develop more suitable RDTs for routine malaria diagnosis in endemic communities.

## Introduction

Malaria is a life-threatening disease that remains a significant global health challenge despite being preventable and treatable. Children are disproportionately affected by malaria, accounting for around 80% of all malaria deaths in sub-Saharan Africa [1]. Malaria in children can present in varying severities, ranging from mild febrile illness to severe malaria, which can result in complications such as cerebral malaria, severe anemia, and, in extreme cases, death. Children, particularly those under the age of five, have underdeveloped immune systems, making them more susceptible to the disease and its complications [2]. Malaria also has long-term consequences in children, such as impairments in cognitive development and physical growth, which can extend into adulthood, affecting educational and socioeconomic outcomes [3]. According to the World Health Organization, in 2023, there were approximately 247 million malaria cases worldwide, with an estimated 619,000 deaths [4]. While significant global efforts have been made to reduce the burden of malaria, progress has slowed in recent years due to various challenges, including drug resistance, the COVID-19 pandemic, and mosquito insecticide resistance [5].

Sub-Saharan Africa bears the highest burden of malaria globally, accounting for about 95% of all malaria cases and 96% of deaths [6]. Children under five years account for around 80% of malaria deaths in the region [7]. Nigeria has a high disease burden, accounting for nearly 27% of global malaria cases and 31% of global malaria deaths [1]. Kano state, in the northern part of Nigeria, is a hotspot for malaria transmission due to its climatic conditions, which favor mosquito breeding and transmission, and socio-economic factors that impede effective healthcare delivery [8]. Given malaria's impact on the broader health system, effective diagnosis is essential to ensure prompt and appropriate treatment, reduce transmission, and ultimately contribute to the elimination goals.

Several factors complicate accurate malaria diagnosis, including overlapping symptoms with other febrile illnesses, such as typhoid, pneumonia, and viral infections [9]. Misdiagnosis often results in inappropriate treatment, which can exacerbate drug resistance and lead to adverse health outcomes. Therefore, precise and timely diagnosis is a cornerstone of effective malaria management and control [10].

Microscopic examination of Giemsa-stained blood smears is widely regarded as the “gold standard” for diagnosing malaria. It is a prerequisite test before the commencement of antimalarial therapy in many developing countries. Improper blood film preparation, germs, fungus, dirt, and cell debris produce false-positive results [11]. Nonetheless, the quality of the stained slide and the competence of the microscopists determine the sensitivity and specificity of microscopy.

Rapid diagnostic test (RDT), an immunochromatographic capture technique, was developed to increase malaria diagnosis sensitivity [12,13]. They detect specific parasite antigens, such as histidine-rich protein 2 (HRP-2), plasmodium lactate dehydrogenase (pLDH), or aldolase [14]. These tests are simple to perform, without complex equipment, and provide results within 15-20 minutes, making them useful in low-resource settings and remote areas. Despite their advantages, the HRP-2-based RDTs, commonly used for detecting Plasmodium falciparum, the most prevalent malaria and lethal species in Africa, have been shown to produce false positives due to persistent parasitemia even after the parasite has been cleared from the bloodstream [15]. Additionally, HRP-2 gene deletions and polymorphisms have been reported in some regions, leading to false negatives and posing a significant challenge to the reliability of RDTs [16].

The polymerase chain reaction (PCR) can identify specific nucleic acid sequences and is valued for its sensitivity to identify pan malaria parasite species or less per microliter of blood. PCR is helpful for both the initial diagnosis of parasites and monitoring drug effects [12,17]. The high initial and overhead costs associated with PCR and the lengthy turn-around time limit its wide use as a point-of-care test for malaria detection in resource-constrained settings [10].

Accurate diagnosis is crucial to effectively manage malaria, as it directly impacts treatment outcomes, disease surveillance, and overall control strategies. This study aims to evaluate the performance of three widely used diagnostic techniques (microscopy, RDTs, and PCR) in diagnosing children with uncomplicated malaria (symptomatic malaria infection with no signs of vital organ disturbance) in Kano, Nigeria.

## Materials and methods

Study area

This cross-sectional study was conducted among febrile children visiting Aminu Kano Teaching Hospital in Kano state, Nigeria, from March 2023 to February 2024. Kano is the second largest city in the country, with a population of close to four million in northern Nigeria. The climatic conditions and mosquito breeding grounds that facilitate the transmission of malaria parasites and malaria are conducive to malaria endemicity, with transmission peaking from September 2023 to February 2024 [8].

Study population

The study included 200 children under 12 years who presented with fever at the Aminu Kano Teaching Hospital, Kano. Eligible children were recruited through convenience sampling. The study's minimum sample size was determined using the sample size formula for a single proportion, with a 32.4% prevalence of malaria parasitemia [8], a precision of 5%, and a standard deviation of 1.96 at 95% confidence intervals. Given a 10% non-response rate, the study required a minimum sample size of 167 participants.

Ethical consideration

Ethical approval was obtained from the Aminu Kano Teaching Hospital Research Ethics Committees, with approval number NHREC/28/01/2020/AKTH/EC/3517, for the original study titled "Molecular Surveillance for HRP2 and HRP3 Gene Deletion in Nigeria from March 2023 to February 2024", where this current paper was generated from. All enrolled participants' samples were processed using PCR, microscopy, and RDT methods as part of the HRP2/3 gene deletion study-approved methodology. Informed consent was sought from parents and legal guardians. Participants who gave their consent were enrolled after being adequately informed about the study objectives, risks, and potential benefits. To ensure confidentiality, the names of the participants were not requested and recorded.

Sample collection and processing

Peripheral blood samples obtained from a finger prick were used to diagnose malaria using the malaria RDT (SD Bioline™) and Giemsa-stained blood smears microscopy. Two trained microscopists viewed each blood film for microscopy. Approximately 100 µL of blood was spotted on the Whatman 3MM filter paper for molecular analysis.

DNA extraction and molecular analysis

DNA was extracted from dried blood spots (DBS) on filter paper using the QIAamp Mini DNA extraction kit (QIAGEN, Inc., San Diego, CA), following the manufacturer's instructions. Sixty microliters of DNA were eluted and stored at -20 °C until further analysis.

Nested PCR targeting the 18S rRNA gene was performed for all extracted DNA using two sets of primers and conditions as previously described [18,19]. All PCR assays were performed using Eppendorf™ Mastercycler X50 thermal cycler using a PCR program that consisted of denaturation at 95 °C for five minutes, followed by 25 cycles (30 cycles in nested) of one minute at 94 °C, two minutes at 60 °C and two minutes at 72 °C, and a final extension period of five minutes at 72 °C. Gel electrophoresis was performed with a 1.2% agarose gel to analyze the PCR products visualized on an ultraviolet (UV) transilluminator (Azure 200, Dublin, CA). Fragment sizes were compared with 100 base pair Solis Biodyne DNA ladder.

Statistical analysis

Sensitivity, specificity, positive predictive value (PPV), and negative predictive value (NPV) calculations were used to assess the diagnostic performance of microscopy and RDT. Cohen's kappa statistic estimated the degree of agreement between the diagnostic tests. The relationship between independent categorical variables was ascertained using the Pearson chi-square test. P-value <0.05 was used to define statistical significance. Statistical analyses were carried out using Statistical Product and Service Solutions (SPSS, version 21.0; IBM SPSS Statistics for Windows, Armonk, NY). To assess the diagnostic performance of the rapid diagnostic test (RDT) and microscopy, a receiver operating characteristic (ROC) curve analysis was performed. The ROC curve was constructed by plotting sensitivity against specificity for both diagnostic methods, with the reference standard being PCR. The area under the curve (AUC) was computed to quantify the overall accuracy of each method, with higher AUC values indicating better diagnostic performance. The ROC curves for RDT and microscopy were compared against the reference line (AUC = 0.5), representing a test with no discriminatory power.

## Results

Demographic characteristics of study participants

**Figure 1:**
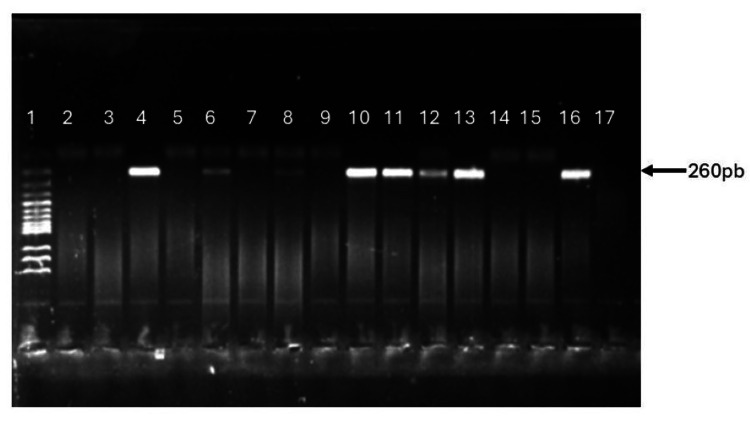
Agarose gel electrophoresis of the human Plasmodium 18s rRNA gene PCR product Lane 1 = 100 bp DNA marker; Lanes 4, 6, 8, 10, 11, 12, and 13 = P. falciparum-infected blood DNA samples; Lanes 2, 3, 5, 7, 9, 14, and 15 = P. falciparum-negative blood DNA samples; Lane 16 = 3D7 (P. falciparum laboratory clone) DNA-positive control; Lane 17 = DNA-negative control

The median age of the 200 children recruited was 9.55 years, with the majority (161, 80.5%) within the 8-12-year age range. The predominant tribe among participants was Fulani, accounting for 109 (54.5%) of the children. Males constituted a slightly higher proportion at 104 (52.0%), compared to 96 (48.0%) females. Ninety-one (45.5%) of the parents/guardians of the recruited children were well-educated up to the tertiary level, while 89 (44.5%) were self-employed. The study reveals a malaria parasite detection rate of 56% (112/200) by microscopy, 30% by RDT (60/200), and 35% (70/200) by nested PCR (Table 1). The PCR product of the 18S rRNA gene visualized under UV light after electrophoresis in 2% agarose gel is presented in Figure 1.

**Table 1:** Socio-demographic distribution and malaria positivity rate of the study participants

Variable	Categories	Frequency (n)	Percent (%)
Age Group	4-7 years	39	19.5
	8-12 years	161	80.5
Ethnicity	Fulani	109	54.5
	Hausa	88	44.0
	Others	3	1.5
Gender	Female	96	48.0
	Male	104	52.0
Parent/Guardian Education Level	Primary	8	4.0
	Secondary	86	43.0
	Tertiary	91	45.5
	Write and read-only	15	7.5
Occupation of the Parent/Guardian	Government employed	56	28.0
	Private employed	53	26.5
	Self-employed	89	44.5
	Unemployed	2	1.0
Malaria Positivity	RDT	60	30.0
	Microscopy	112	56.0
	PCR	70	35.0

Distribution of malaria diagnosis test results based on possible risk factors

The distribution of malaria test results for microscopy, RDT, and nested PCR based on some tested possible risk factors are shown in Table 2. Children aged 8-12 years showed a higher malaria positivity rate across all testing methods, with PCR producing a significant effect (p = 0.012). All tests indicated a higher malaria prevalence among male children, though this difference was not statistically significant. Similarly, more positive cases were observed among children living in rural areas without statistical significance. Children with stagnant water in their neighborhoods had significantly higher malaria prevalence across all tests (microscopy, p < 0.000; RDT, p < 0.000; PCR, p = 0.0002). Those who played outside at night showed higher positivity rates for microscopy and RDT but lower rates for PCR (p = 0.042). Additionally, children presenting with fever were significantly associated with positive outcomes for RDT (p < 0.000) and PCR (p = 0.046).

**Table 2:** Distribution of malaria diagnosis test results based on socio-demographics and possible risk factors (n=200) Data are presented as n (%); * Significant at p<0.05. PCR = polymerase chain reaction; RDT = rapid diagnostic test

Characteristics	Microscopy		RDT		PCR	
	Positive n (%)	p-value	Positive n (%)	p-value	Positive n (%)	p-value
Age						
4-7	21(18.8%)	0.762	14 (23.3%)	0.483	7 (10%)	0.012*
8-12	91(81.3%)		46 (76.7%)		63 (90%)	
Gender						
Male	58 (51.8%)	0.286	31 (51.7%)	0.599	36 (51.4%)	0.572
Female	54 (48.2%)		29 (48.3%)		34 (48.6%)	
Residence						
Urban	25 (22.3%)	0.930	16 (26.7%)	0.533	13 (18.6%)	0.579
Rural	87 (77.7%)		44 (73.3%)		57 (81.4%)	
Always playing outside at night						
Yes	64 (57.1%)	0.977	38 (63.3%)	0.349	33 (47.1%)	0.042*
No	48 (42.9%)		22 (36.7%)		37 (52.9%)	
Use insecticide net						
Yes	63 (56.3%)	0.282	35 (58.3%)	0.875	37 (52.9%)	0.173
No	49 (43.8%)		25 (41.7%)		33 (47.1%)	
Live near stagnant water						
Yes	71 (63.4%)	< 0.000*	42 (70%)	< 0.000*	38 (54.3%)	0.0002*
No	41 (36.6%)		18 (30%)		32 (45.7%)	
Last malaria episodes						
< 1 month	92 (82.1%)	0.625	52 (86.7%)	0.596	55 (78.6%)	0.480
2-6 months	18 (16.1%)		8 (13.3%)		13 (18.6%)	
>6 months	2 (1.8%)		0		2 (2.9%)	
Drug used						
ACT	41 (36.6%)	0.700	24 (40%)	0.392	25 (35.7%)	0.980
Chloroquine	12 (10.7%)		5 (8.3%)		7 (0.1%)	
SP	47 (42%)		28 (46.7%)		31 (44.3%)	
Herbs	11 (9.8%)		2 (3.3%)		6 (0.1%)	
Other drugs	1 (0.9%)		1 (1.7%)		1	
Test before treatment						
Yes	37 (33%)	0.033*	32 (53.3%)	0.018*	22 (31.4%)	0.096*
No	75 (67%)		28 (46.7%)		48 (68.6%)	
Fever presentation						
Yes	82 (73.2%)	0.953	59 (95.0%)	<0.000*	45 (64.3%)	0.046*
No	30 (26.8%)		1 (5.0%)		25 (35.7%)	

Performance of the different diagnostic assays used for evaluating malaria positivity

**Figure 2:**
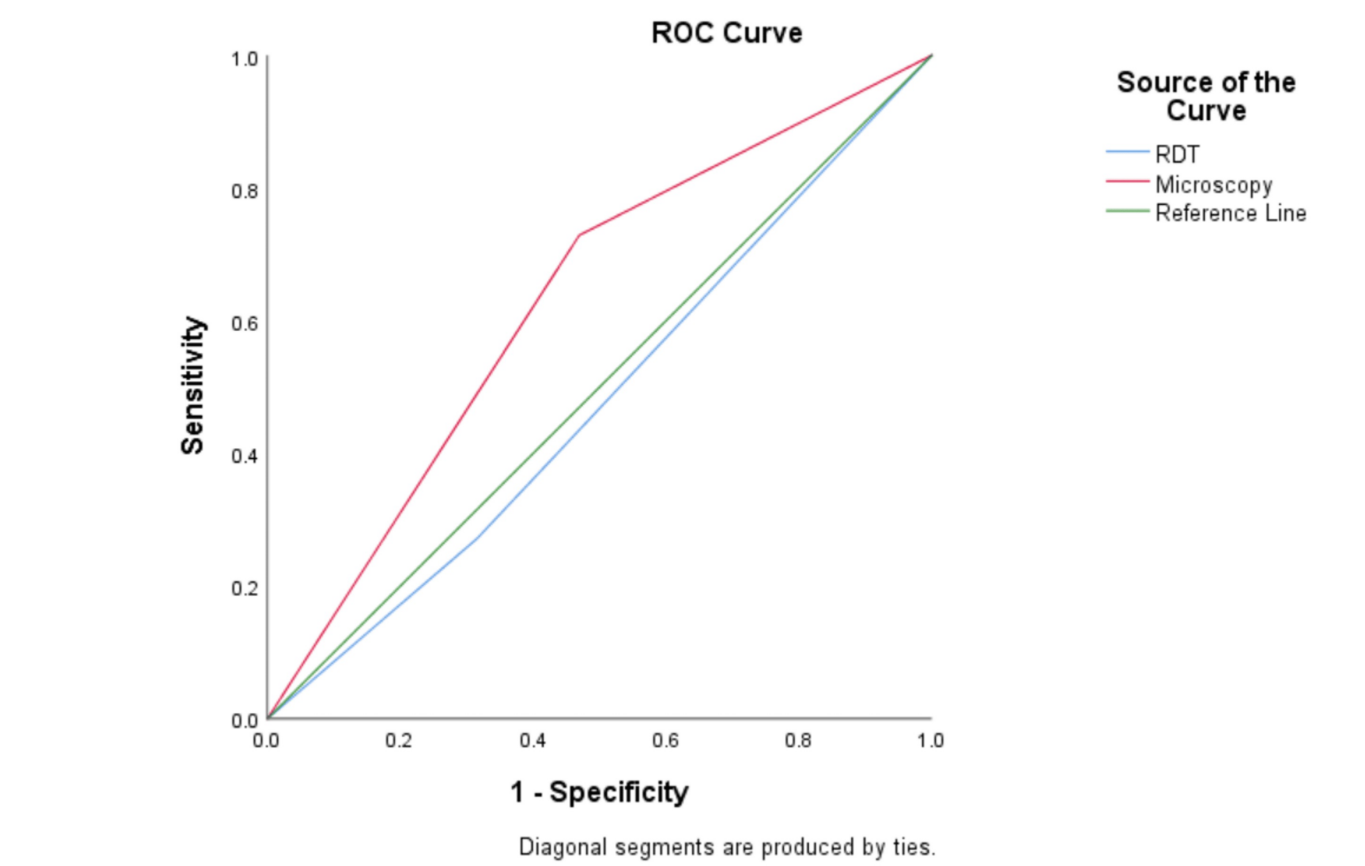
Receiver operating characteristic (ROC) curve analysis of malaria diagnostic techniques ROC for microscopy and rapid diagnostic test (RDT) versus polymerase chain reaction (PCR) as the reference method

Table 3 shows the performance of RDT and microscopy for evaluating malaria positivity, using nested PCR as the reference standard. The RDT shows low sensitivity (27.1%) compared to microscopy and low PPV (31.5%), indicating that it misses many true positive cases. Its high specificity (68.0%) suggests some ability to identify true negative cases correctly. Microscopy has significantly higher sensitivity (72.9%) compared to RDT. However, its specificity is relatively lower (53.0%) but with a higher negative predictive value (78.0%). A significant difference (p = 0.0006) was observed between the performance of RDT and microscopy using PCR as the test standard. The ROC curve analysis revealed that the AUC for RDT did not differ significantly against PCR (AUC: 0.48; p = 0.61), while microscopy showed a significant difference (AUC: 0.63; p = 0.003) against PCR as a reference standard (Figure 2). 

**Table 3:** Test performance of the different diagnostic assays used for evaluating malaria positivity *Significant p<0.05 Pos = positive; Neg = Negative; PPV = positive predictive value; NPV = negative predictive value; p-value* = p value for specificity and sensitivity; p-value# = p value for ROC curve

PCR			Sensitivity (95% CI)	Specificity (95% CI)	PPV (95% CI)	NPV (95% CI)	p-value	AUC (95% Cl)	p-value^#^
Test	Pos	Neg							
RDT									
Pos	19	41	27.1 (0.17-0.39)	68.0 (0.60-0.76)	31.5 (0.20-0.45)	64.0 (0.55-0.72)	0.63	0.48 (0.39-0.56	0.61
Neg	51	89							
Microscopy									
Pos	51	61	72.9 (0.61-0.83)	53.0 (0.44-0.62)	46.9 (0.36-0.55)	78.0 (0.68-0.86)	0.0006*	0.63 (0.55-0.71)	0.003*
Neg	19	69							

## Discussion

This study analyzes three diagnostic tools often used in routine and research settings to detect malaria infection among children in Kano state, Nigeria. The results reveal distinct strengths and limitations of each diagnostic method, with significant implications for malaria control strategies in endemic regions.

Our results showed a 35% malaria prevalence rate using PCR, while microscopy and RDT detected 56% and 30% of cases, respectively. The higher microscopy detection rate compared to PCR indicates greater sensitivity in this study context. However, microscopy produced a high number of false positives (46.9%), raising questions about its specificity. The relatively high false-positive rate could be attributed to various factors, including poor blood film preparation, artifacts, or misinterpretation by microscopists. These findings emphasize the importance of regular quality assurance and training for laboratory personnel to improve diagnostic accuracy. Low parasite density in some participants may explain why microscopy failed to identify approximately 27% of the individuals [20]. This group of untreated individuals poses a significant challenge to malaria eradication efforts because they eventually serve as infection reservoirs for ongoing transmission [13].

In contrast to microscopy, RDTs showed a lower sensitivity of 27% but a higher specificity of 68%. Due to their low sensitivity, RDTs may have missed a good percentage of malaria cases. Similar to a study done in southeast Nigeria [21], RDT had more false-negative results but fewer true- and false-positive ones. Nonetheless, this study's false positivity rate is less than that of a study carried out in southwest Nigeria [22]. Unfortunately, false positivity or negativity frequently results in misdiagnosis because RDT and microscopy examination are the only bases for treatment in clinical settings. The ROC curve analysis was employed to determine the AUC, which ranged from 0.5 to 1, with 0.5 indicating a poor test performance and 1 representing an exceptional test performance. AUC values of RDT (0.48, 0.39-0.56) and microscopy (0.63, 0.55-0.71) suggested carefully using these conventional diagnostic methods in malaria screening. AUC data suggested that RDT and microscopy in comparison to PCR were not very effective in discriminating malaria-positive and malaria-negative individuals. However, microscopy has been shown to perform better compared to RDT.

Several factors may be responsible for the low sensitivity and specificity observed using RDT to diagnose malaria in this study. RDTs that detect P. falciparum-specific HRP2 are the most used due to their efficiency, high thermal stability, sensitivity, specificity, cost-effectiveness, and easy-to-use abilities [23]. Since P. falciparum HRP-2 antigen remains in the bloodstream even after treatment and parasite clearance, RDT cannot differentiate between passive and active infection, which could result in false-positive results from RDT [24]. Furthermore, the RDT is prone to genetic mutations and deletions, as observed in recent years [16,25]. Patients infected with P. falciparum with a deletion in the HRP2/3 gene locus would remain undetected, resulting in false negatives, and remain untreated. These findings are consistent with reports from other endemic regions, where HRP2/3 deletions have contributed to increased false-negative rates [26]. The persistence of HRP-2 antigen in the bloodstream, even after parasite clearance, further complicates the interpretation of RDT results, potentially leading to overdiagnosis in some cases. Such limitations highlight the challenges of relying solely on RDTs for routine malaria diagnosis and treatment decisions in endemic areas such as Kano.

Considering PCR as the reference, microscopy identified more positive cases but also had more false positives of 61 (30.5%). PCR’s ability to be precise, detect low-level parasitemia, and differentiate between Plasmodium species makes it a valuable tool for accurate malaria diagnosis. However, the practical application of PCR in resource-limited settings is hindered by its high cost, technical complexity, and longer turnaround time. This limitation restricts its use primarily to research settings or as a reference standard rather than a routine diagnostic tool. Nevertheless, PCR remains critical for validating other diagnostic methods and detecting submicroscopic infections that could serve as reservoirs for ongoing transmission [27].

Furthermore, specific risk factors, such as age, playing outside at night, and fever presentation, were significantly associated with positive PCR results, indicating that demography, behavioral, and environmental factors play a critical role in malaria transmission in this study. This suggests that diagnostic strategies should also incorporate risk factor assessments to identify high-risk individuals who may benefit from more sensitive testing methods such as PCR [28]. Public health interventions targeting behavioral modifications, such as using insecticide-treated nets and limiting outdoor activities during peak mosquito biting times, could further reduce malaria transmission.

Variations in parasite density, the presence of mixed infections, and the persistence of parasite antigens in the blood likely contributed to the discrepancies observed between diagnostic tools in this study. These factors underscore the need for ongoing research to refine RDTs and enhance their sensitivity and specificity, particularly in detecting P. falciparum strains with HRP2/3 deletions. The increasing frequency of pfhrp2 and pfhrp3 gene deletions poses a growing threat to malaria control efforts, leading to false-negative results in RDTs. Studies in Asia have documented P. falciparum strains lacking pfhrp2, while Eritrea has reported the highest prevalence of pfhrp2/3 deletions in Africa, leading to a shift toward non-HRP2-based RDTs. Although lower proportions of pfhrp2/3 deletions have been observed in field isolates from East and West Africa, the issue remains a concern. Limited studies in Nigeria have assessed pfhrp2/3 deletions, with findings suggesting a low risk of false-negative RDT results due to these deletions [16,29]. However, as this situation could change quickly, continuous monitoring is essential to ensure that RDTs remain a reliable tool in malaria diagnostics. Developing new diagnostic tools that combine the speed and simplicity of RDTs with the sensitivity of molecular techniques such as PCR would significantly advance malaria diagnosis [30].

One of the main limitations of this study is the relatively small sample size, which may affect the generalizability of the findings. Additionally, only one type of RDT was used, which may not reflect the performance of other commercially available RDTs. Future studies should include larger sample sizes and multiple RDT brands and assess the impact of seasonal variation on diagnostic performance.

## Conclusions

In conclusion, this study provides valuable insights into the comparative effectiveness of microscopy, RDT, and PCR for malaria diagnosis in children in Kano, Nigeria. While microscopy remains the gold standard for diagnostic accuracy, its reliability is highly dependent on the quality of laboratory facilities and the expertise of personnel, which may impact its consistency. To enhance diagnostic accuracy and reduce the risk of misdiagnosis in low-resource areas, a combined approach using microscopy and RDTs is recommended. These findings add to the growing body of evidence for refining malaria diagnostic strategies and highlight the urgent need for more reliable, cost-effective diagnostic tools in malaria-endemic regions.

## Appendices

**Table 4:** Rationale for collaboration

Author Name & Institution	Rationale
Salma A.R. Suliman, Institute of Endemic Diseases, University of Khartoum, Khartoum, Sudan	There are existing collaborations between the Humboldt Research Hub-Center for Emerging and Re-emerging Infectious Diseases (HRH-CERID), Ladoke Akintola University of Technology (LAUTECH), Ogbomoso, Nigeria, and the Institute of Endemic Diseases, University of Khartoum, Khartoum, Sudan, Within the grant awarded for establishing HRH-CERID, LAUTECH, the institute of endemic diseases, the University of Khartoum is the African collaborator. The Author listed with this affiliation, Salma A.R. Suliman, is currently undertaking her Ph.D. research at HRH-CERID, LAUTECH.
Prof Bolaji Thomas of the Department of Biomedical Sciences, College of Health Sciences and Technology, Rochester Institute of Technology, Rochester, USA	Prof Bolaji Thomas of the Department of Biomedical Sciences, College of Health Sciences and Technology, Rochester Institute of Technology, Rochester, USA, has been a long-standing collaborator. We have collaborated on over 20 research projects and published over 20 papers. He is also a Ph.D. co-supervisor of Salma Suliman and Adedolapo Olorunfemi
Mr. Abdulmalik Ahmed Sani, Department of Life Sciences Kano State Polytechnic, Kano State, Nigeria	Mr. Abdulmalik Ahmed Sani is one of our collaborators in a nationwide study investigating the HRP2/3 deletion. He spearheads the collection of samples for this study.
